# Meningeal Infiltration of the Spinal Cord by Non-Classically Activated B Cells is Associated with Chronic Disease Course in a Spontaneous B Cell-Dependent Model of CNS Autoimmune Disease

**DOI:** 10.3389/fimmu.2015.00470

**Published:** 2015-09-15

**Authors:** Amy K. Dang, Yodit Tesfagiorgis, Rajiv W. Jain, Heather C. Craig, Steven M. Kerfoot

**Affiliations:** ^1^Department of Microbiology and Immunology, Schulich School of Medicine and Dentistry, Western University Canada, London, ON, Canada

**Keywords:** B cells, EAE, demyelination, inflammation, meninges

## Abstract

We characterized B cell infiltration of the spinal cord in a B cell-dependent spontaneous model of central nervous system (CNS) autoimmunity that develops in a proportion of mice with mutant T and B cell receptors specific for myelin oligodendrocyte glycoprotein. We found that, while males are more likely to develop disease, females are more likely to have a chronic rather than monophasic disease course. B cell infiltration of the spinal cord was investigated by histology and FACs. CD4^+^ T cell infiltration was pervasive throughout the white and in some cases gray matter. B cells were almost exclusively restricted to the meninges, often in clusters reminiscent of those described in human multiple sclerosis. These clusters were typically found adjacent to white matter lesions and their presence was associated with a chronic disease course. Extensive investigation of these clusters by histology did not identify features of lymphoid follicles, including organization of T and B cells into separate zones, CD35^+^ follicular dendritic cells, or germinal centers. The majority of cluster B cells were IgD^+^ with little evidence of class switch. Consistent with this, B cells isolated from the spinal cord were of the naïve/memory CD38^hi^ CD95^lo^ phenotype. Nevertheless, they were CD62L^lo^ and CD80^hi^ compared to lymph node B cells suggesting that they were at least partly activated and primed to present antigen. Therefore, if meningeal B cells contribute to CNS pathology in autoimmunity, follicular differentiation is not necessary for the pathogenic mechanism.

## Introduction

The best evidence supports the hypothesis that multiple sclerosis (MS) is an autoimmune disease of the central nervous system (CNS) driven by immune cells targeting myelin antigens. The autoimmune response results in chronic inflammation of the CNS, demyelination, destruction of axons, and neurodegeneration over an extended period of time (1, 2). The presence of infiltrating immune cells in MS CNS tissues, including potentially myelin-specific T and B cells, supports a primary immune etiology for disease (3–6), as do studies that have identified genes associated with the immune system, immune regulation, and antigen presentation as the primary genetic risk factors for MS (7). Deliberate induction of myelin-targeting autoimmunity in animal models results in CNS inflammation and pathology that recapitulates some features of human MS, although the degree to which these models resemble human disease varies depending on how autoimmunity is induced and on the species or strain of animal used (8–10).

Outside of the laboratory, the vast majority of immune responses incorporate antigen targeting by multiple subpopulations of both T and B cells. However, in recent decades, research interest has focused heavily on CD4^+^ T cells and this is particularly true of MS research. Considerable advances in our understanding of how B cells collaborate with CD4^+^ T cells during the initiation and development of a response highlight the importance of understanding immune responses as a whole. When both T and B cells are involved in target recognition, germinal center (GC) formation is the typical result. GCs are the source for long lived, high affinity B cell and antibody responses fundamental to normal, complex protective, and pathogenic immunity (11). While best known for their role in antibody production, it is becoming increasingly apparent that B cells are also important regulators and modulators of the immune response through the production of cytokines (12, 13) and presentation of antigen to T cells (14). As antigen-presenting cells (APCs), B cells very efficiently take up antigen that binds to their specific B cell receptor (BCR) and process it for presentation to T cells. The resulting “cognate” interactions between T and B cells specific for the same or physically linked antigen are foundational to the development of most complex immune responses (15, 16), almost certainly including those underlying organ-specific autoimmune diseases like MS (4).

Considerable evidence supports an important role for B cells in addition to T cells in driving MS pathology. The presence of antibodies in the cerebrospinal fluid (CSF) and B cells in the CNS infiltrate has long been recognized as features of disease (4, 5). More recently, targeted depletion of CD20-expressing B cells using humanized anti-CD20 antibodies was shown to very effectively reduce inflammatory signs and relapses in MS (17). This ignited interest in B cells as therapeutic targets. However, a more recent trial of a soluble recombinant version of the cytokine receptor TACI (TACI-Fc), which depletes B cells through the inhibition of the cytokines BAFF and APRIL, was halted early due to indications that treatment increased relapse rate (18). Anti-CD20 and TACI-Fc deplete B cells through very different mechanisms and target different subsets of cells (19). CD20 is not expressed by antibody-producing plasma cells and therapeutic benefit of anti-CD20 was observed well prior to any reduction in antibody levels. By contrast, TACI-Fc does target plasma cells in addition to mature B cells. Further, as both T cells and neurons express receptors for BAFF (20, 21), the effects of this drug likely extend well beyond B cell depletion. Therefore, while antibodies may contribute to pathology (1), the primary B cell contribution to MS is through some other mechanism(s), perhaps via APC function or cytokine modulation of the autoimmune response. Furthermore, the effectiveness of B cell depletion via anti-CD20 (17) suggests that their pathogenic role is ongoing and drives chronic disease. The lack of benefit and perhaps pathogenic effects of TACI-Fc confuses the issue, and highlights the need to identify pathogenic and protective B cell subpopulations and their roles in CNS autoimmunity.

It is also not clear where, anatomically, B cells exert their pathogenic function. There has been considerable recent interest in clusters of B and T cells observed in the meninges in post-mortem studies of MS brains, often in direct association with demyelinating lesions (3, 6, 22). Some studies have focused on the potential similarity of these structures to secondary lymphoid organs, suggesting that they may perform similar functions in propagating immune responses from within the inflamed CNS (23, 24). Understanding the pathogenic contributions of B cells and the role played by meningeal clusters to ongoing disease will require models that appropriately recapitulate a complex anti-myelin immune response.

Animal models with induced anti-myelin autoimmunity are referred to by the umbrella term “experimental autoimmune encephalomyelitis” (EAE). Currently, the most commonly used versions of this model are induced through immunization with short peptides mimicking dominant CD4^+^ T cell epitopes derived from myelin protein antigens. By their design, these models severely limit the involvement of other lymphocytes, including B cells that would normally participate in antigen targeting. Immunization with larger protein antigens can overcome this limitation (25). Alternatively, B cells may contribute to several non-immunization-based models. CNS autoimmunity can develop “spontaneously” (sEAE) in a proportion of mice with enhanced anti-myelin activity due to expression of mutant antigen-specific receptors (26–28). In most cases, enhanced anti-myelin immunity is restricted to T cells. However, two groups (29, 30) independently reported that disease occurs with much greater incidence in mice expressing both a transgenic T cell receptor (TCR) specific for myelin oligodendrocyte glycoprotein (MOG)_35–55_ peptide (26) and a BCR heavy chain knock-in mice that, when paired with an appropriate light chain, also confers specificity for MOG protein on ~20% of B cells (31). This model, with predetermined B as well as T cell recognition of the myelin autoantigen, may therefore be valuable for investigations of the B cell collaboration with autoimmune T cells to promote CNS autoimmune disease.

Here, we characterize B cells participating in spontaneous CNS autoimmune disease in 2D2 IgH^MOG^ mice. We observe that sEAE can follow either a monophasic or chronic disease course and that this correlates with ongoing inflammation in the spinal cord and with formation of meningeal clusters of T and B cells in particular. However, we found little evidence that the B cells in meningeal clusters are activated in a conventional sense. Finally, only in a very rare case did we find any evidence of development of follicular features in meningeal clusters, indicating that if clusters do contribute to ongoing pathology in CNS autoimmunity, the relatively unorganized form must represent the minimum requirement for disease.

## Materials and Methods

### Mice

Wild type C57Bl/6 and 2D2 TCR transgenic (26) mice were purchased from Jackson Laboratories. IgH^MOG^ MOG-specific BCR knock-in mice (32) were received as a gift from Dr. Hartmut Wekerle. Genotyping was accomplished using the following primers: 2D2: f-GCG GCC GCA ATT CCC AGA GAC ATC CCT CC, r-CCC GGG CAA GGC TCA GCC ATG CTC CTG; IgH^MOG^: f-GGA TTG CAC GCA GGT TCT CCG, r-CCG GCC ACA GTC GAT GAA TCC. All mice were housed under specific pathogen-free conditions at the West Valley Barrier Facility at Western University Canada. Animal protocols (#2011-047) were approved by the Western University Animal Use Subcommittee.

### Antibodies for flow cytometry and histology

The following antibodies were purchased from BD Biosciences: anti-CD4-V450 (RM4-5), anti-CD45R-V450 (RA3-6B2), anti-CD45R-APC-Cy7 (RA3-6B2), anti-CD45R-A647 (RA3-6B2), anti-CD138-BV421 (281-2), anti-CD19-BV711 (1D3), anti-CD95-PE-Cy7 (Jo2), anti-Bcl6-A647 (K112-91), anti-IgG1-APC (A85-1), anti-CD62L-A700 (MEL-14), anti-CD35-biotin (8C12), anti-CD49D (R1-2), anti-CD62P (RB40.34), anti-IgM-APC (II/41), and anti-CD80-PE (16-10A1). The following antibodies were purchased from BioLegend: anti-CD4-A647 (RM4-5), anti-CD3e-FITC (145-2C11), and anti-rabbit DyLight 649 (Poly4064). The following antibodies were purchased from eBioscience: anti-CD4-PE-Cy5 (RM4-5), anti-PNAd-A488 (MECA-79), anti-CD38-PE (90), anti-IgM-PE-Cy5 (II/41), anti-IgD-APC (11-26c), anti-IgD eF450 (11-26c), anti-CD279-Biotin (RMP1-30), anti-CD273-Biotin (TY25), anti-F4/80-Biotin (BM8), Streptavidin-APC-eF780, and Streptavidin-APC. Anti-Ki-67 (SP6) unconjugated was purchased from Thermo Scientific. FluoroMyelin Red for myelin staining was purchased from Invitrogen.

### Spontaneous 2D2 IgH^MOG^ EAE model

2D2^+/−^ IgH^MOG+/−^ double mutant mice were generated as the F1 generation of 2D2^+/−^ mice crossed with IgH^MOG+/+^ mice. Where indicated, some mice received a single i.v. injection of 250 ng pertussis toxin (PTX – List Biological Laboratories, Inc.) between 31 and 33 days of age. Clinical disease was monitored daily and was scored as follows: 0, no clinical signs; 1, tail paralysis; 2, tail paralysis and hind limb weakness; 3, hind limb paralysis; and 4, complete hind limb paralysis and front limb weakness. Half points were given for intermediate scores.

### Flow cytometry

Flow cytometry analysis of T cells and B cells harvested from mouse lymph nodes and spinal cords was performed, as previously described (25). Briefly, spinal cord and lymph nodes, including inguinal, axillary, and cervical lymph nodes, were harvested from mice after perfusion with ice cold PBS. Individual spinal cords were additionally dissociated through a wire mesh after which myelin was removed using a Percoll (GE Healthcare Life Sciences) gradient. Leukocytes were collected at the 37/90% Percoll interface.

Both lymph node and isolated spinal cord cell suspensions were blocked with an anti-Fc-γ receptor (CD16/32 2.4G2) in PBS containing 1% FBS before further incubation with the listed combination of staining antibodies. Dead cells were excluded by staining with the Fixable Viability Dye eFluor506 (eBioscience). Flow cytometry was performed on a LSRII cytometer (BD Immunocytometry Systems) and analyzed with FlowJo software (Treestar).

### Immunofluorescent histology

Spinal cords and lymph nodes were extracted from mice and prepared, as previously described (25). Briefly, whole lymph nodes and spinal cords were fixed in periodate–lysine–paraformaldehyde (PLP) and subsequently passed through sucrose gradients to protect from freezing artifacts. Lymph nodes were frozen whole in OCT (TissueTek) media. Spinal cords were cut into five to nine evenly spaced pieces and arranged in order prior to freezing in OCT. Serial cryostat sections (7 μm) were blocked in PBS containing 1% Bovine Serum Albumin, 0.1% Tween-20, and 10% rat serum before proceeding with staining. Sections were mounted with ProLong Gold Antifade Reagent (Invitrogen) and stored at −20°C. Tiled images of whole spinal cord sections (20×) were imaged using DM5500B fluorescence microscope (Leica).

### Image and statistical analyses

The size of meningeal clusters in images of diseased spinal cords was analyzed using ImageJ software. PRISM software was used for all statistical analysis. Unless otherwise stated, single comparisons were performed using a Student’s *t*-test and multiple comparisons were performed by ANOVA followed by a Tukey *post hoc* test.

## Results

### Disease incidence in 2D2 IgH^MOG^ double mutant mice

**Figure 1:**
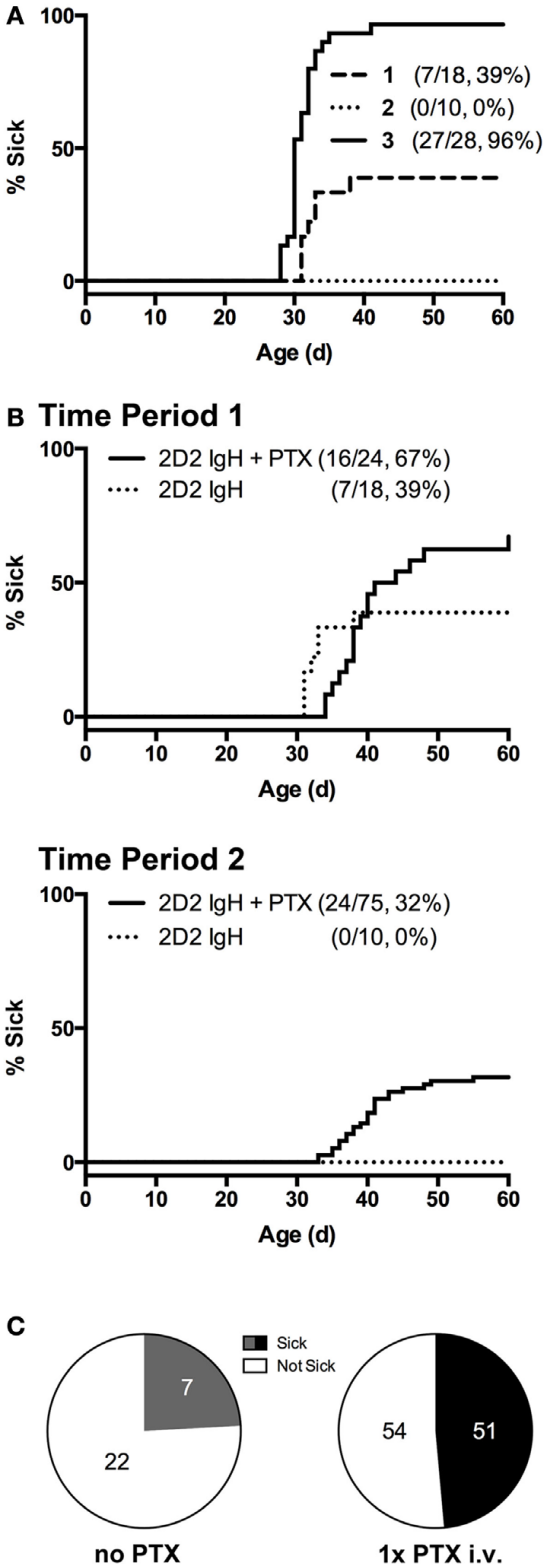
**Incidence of spontaneous CNS autoimmune disease (sEAE) in 2D2 IgH^MOG^ mice**. **(A)** Disease onset curves for three representative sequential 4- to 6-month time-periods (Timepoint 1, 2, and 3) selected from the ~2-year period of study. The percent of mice in each group to demonstrate signs of disability as determined by the disease scoring system (see Materials and Methods) is shown (% Sick) **(B,C)** PTX administration increases disease incidence. **(B)** Single injections of 250 ng PTX i.v. were administered to ~32 days old 2D2 IgH^MOG^ mice, which were subsequently followed for onset of disease compared to unmanipulated mice. **(C)** Fraction of diseased mice in PTX-untreated and -treated mice, restricted to times when the overall incidence was below 80%. Significantly more PTX-treated mice developed disease as determined by Chi-square analysis (*p* = 0.0003, df = 13.13,1).

We followed mice bearing mutant TCR and BCR specific for MOG autoantigen for the development of CNS autoimmune disease. Mice demonstrating overt signs of physical disability were defined as “sick.” Consistent with the previous descriptions (29, 30, 33), a proportion of unmanipulated 2D2^+/−^ IgH^MOG+/+^ mice (here after described as 2D2 IgH^MOG^) developed sEAE (Figure 1A). No disease was observed in either 2D2 (TCR) or IgH^MOG^ (BCR) single mutant mice (Not Shown); it is clearly demonstrating that antigen recognition by both T and B cells contributes to disease development in double mutant mice. Interestingly, males were significantly more likely to develop disease than females, although there was no difference in the time of onset (Table 1). Although previous studies did not note gender differences, the incidence data presented by Krishnamoorthy et al. (30) suggest a similar trend in male bias.

**Table 1:** **Disease profiles in 2D2 IgH^MOG^ mice by gender**.

	*n*=	Sick	Incidence	Day onset	SEM	Max score	SEM
Male	154	95	61.7%	36.7	(±0.52)	2.55	(±0.107)
Female	137	68*	49.6%	36.6	(±0.68)^n.s.^	2.43	(±0.143)^n.s.^

* = 0.0387, df = 4.275, 1 by Chi squared analysis. Note that mice that did not develop disease were not included in analysis of onset and severity.

*n.s., not significant*.

Overall incidence was highly variable over the study period. Initially, 39% of unmanipulated mice developed signs of disease (Figure 1A, Timepoint 1), but over ~2 years of study incidence fell to 0% (Timepoint 2) but later rose to nearly 100% incidence (Timepoint 3). Season has previously been identified as a factor contributing to susceptibility to EAE in a different induced model (34), but did not explain the variance observed in the case of our 2D2 IgH^MOG^ colony. We also excluded obvious changes in environmental factors, such as food or alterations in animal care. Differences in animal housing, largely attributed to differences in microbial exposure, are well known to impact EAE models and spontaneous models, in particular, both between institutions and within the same colony (27, 35). We did not investigate commensal bacteria in our own studies, but unexplained changes in microbiota over time may be the underlying reason for the dramatic shifts in incidence we observed within our colony. Nevertheless, this suggests that, like human MS, spontaneous CNS autoimmune disease in 2D2 IgH^MOG^ mice is variable and influenced by environmental factors.

Pertussis toxin (PTX) is commonly used in the induction of several models of immunization-induced EAE, particularly in C57Bl/6 mice. While the disease-promoting mechanism(s) are not entirely clear (36–38), PTX represents an antigen non-specific pathway to promote disease. Indeed, PTX was shown to increase incidence in a similar model of otherwise spontaneous CNS autoimmunity that develops in mice expressing a transgenic TCR to myelin basic protein (27). In our hands, we similarly found that a single i.v. injection of 250 ng PTX was sufficient to significantly increase disease incidence in 2D2 IgH^MOG^ mice during periods of lower disease incidence (<80%) (Figures 1B,C). Disease in PTX-treated mice was otherwise indistinguishable from that in mice that did not receive PTX (not shown). The mechanism by which it promotes disease induction is not yet clear, but we (37) and others (36, 39) have shown that PTX has innate immunomodulatory effects. Therefore, PTX may act as a surrogate for environmental factors that promote development of CNS autoimmune disease.

### 2D2 IgH^MOG^ mice develop either monophasic or chronic disease

**Figure 2:**
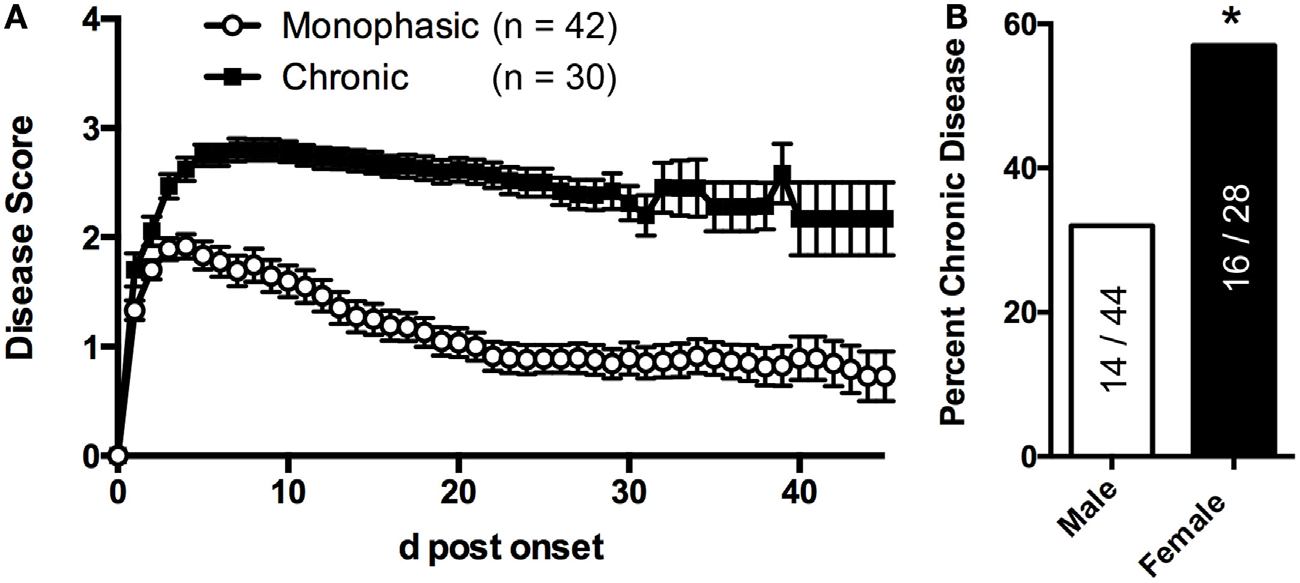
**sEAE in 2D2 IgH^MOG^ mice can follow a monophasic or chronic disease course**. **(A)** 2D2 IgH^MOG^ mice were allowed to develop CNS autoimmune disease and severity was evaluated daily. Mice that developed signs of CNS autoimmune disease for at least 21 days (excluding mice that had been used experimentally or were euthanized prior to 21 days) were divided into “monophasic” or “chronic” categories based on the following criteria: chronic – no more than 1 point recovery after the acute phase as determined by the standard 5 point score system. Monophasic – recovery of at least 1 point on the severity scale and a final score <2. **(B)** Compared to male mice, a greater proportion of female mice develop chronic disease as determined by Chi-square analysis (*p* = 0.0004, df 12.65,1).

2D2 IgH^MOG^ mice were evaluated daily for disease severity. Of those that showed signs of disease (defined as “sick”), the majority of mice had severe disability of the tail, hindlimbs, and partial involvement of the forelimbs, reminiscent of other EAE models and consistent with previous descriptions of this model (29, 30). Unlike for disease incidence (see above), there was no difference between males and females in maximum disease severity (Table 1). We observed that disease typically followed one of two courses; after the initial acute phase some mice largely recovered with little evidence of ongoing disability while others showed little sign of recovery. Therefore, we grouped mice that survived past 21 days post disease onset (i.e., that had not been used experimentally or been euthanized early due to severe disease) into one of two groups based on their final disease status: (1) chronic – mice with no more than 1 point recovery after the acute phase as determined by the standard 5 point score system, and 2) Monophasic – mice that recovered at least 1 point on the severity scale and had a final score <2 (Figure 2). Further evaluation of these populations showed that while the timing of disease onset was not different between them, the chronic group attained a significantly higher maximum disease score (Table 2). This indicates that while the populations were separated into the chronic or monophasic groups based principally on their status at the end of the study, the differences between groups manifested themselves earlier in the acute phase of disease (Figure 2A). Interestingly, while males were more likely to develop disease (see above), females were more likely to have a chronic disease course (Figure 2B).

**Table 2:** **Monophasic and chronic disease profiles in 2D2 IgH^MOG^ mice**.

	*n*=	Day onset	SEM	Max score	SEM
Monophasic	42	38.2	(±0.62)	2.26	(±0.117)
Chronic	30	35.8	(±1.47)^n.s.^	2.98	(±0.106)***

*** < 0.001.

*n.s., not significant*.

### Characterization of the B cell response in 2D2 IgH^MOG^ mice

**Figure 3:**
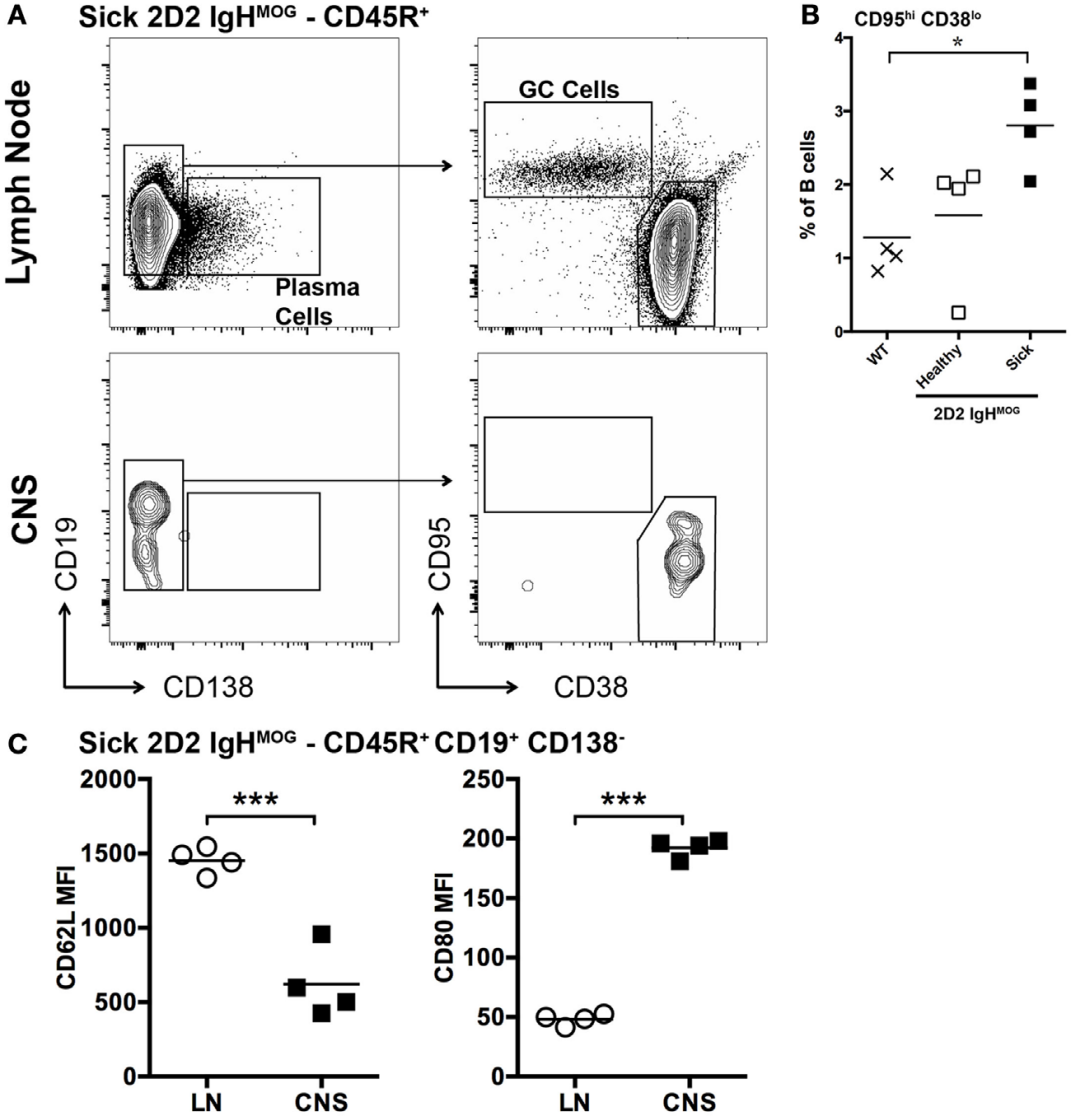
**Characterization of B cells in the lymph nodes and spinal cords of 2D2 IgH^MOG^ mice with sEAE**. **(A)** Lymph nodes and spinal cords were harvested from healthy wild type mice as well as 2D2 IgH^MOG^ mice that had either developed disease (sick – 20–30 days post onset) or not (healthy – age matched). Cells were prepared and analyzed by FACS. CD45R^+^ cells were first selected. An example of the gating strategy to identify Plasma cells [**(A)** – left] and B cells with a GC phenotype [**(A)** – right] from the CD45R^+^ pool is shown for lymph node and spinal cord cells isolated from a single sick mouse. **(B)** Quantification of CD95^hi^ CD38^lo^ germinal center B cells in the lymph nodes of wild type and healthy or sick 2D2 IgH^MOG^ mice. Each symbol represents an individual mouse. *p < 0.05. **(C)** Comparison of CD62L (left) and CD80 (right) expression by CD45R^+^ CD19^+^ CD138^−^ B cells isolated from lymph nodes or the CNS of the same mouse. Each symbol represents an individual sick mouse. ***p < 0.001 as determined by paired Student’s *t*-test. One representative of two experiments shown.

Previous studies employing the 2D2 IgH^MOG^ model focused primarily on T cell activation (29, 30, 33) and information about B cell activation in this or other spontaneous models is very limited. Immune responses that incorporate both T and B cell recognition of antigen typically result in a GC response. Consistent with this, significantly more CD95^hi^ CD38^lo^ GC B cells were present in lymph nodes harvested from sick 2D2 IgH^MOG^ mice (~3 weeks post onset) compared to wild type or age-matched 2D2 IgH^MOG^ that did not develop sEAE (Figures 3A,B). It should be noted that, with disease progression and severity, we observed lymph node atrophy and in some cases little remaining GC response could be detected (not shown).

### Evaluation of spinal cord pathology in 2D2 IgH^MOG^ mice

**Figure 4:**
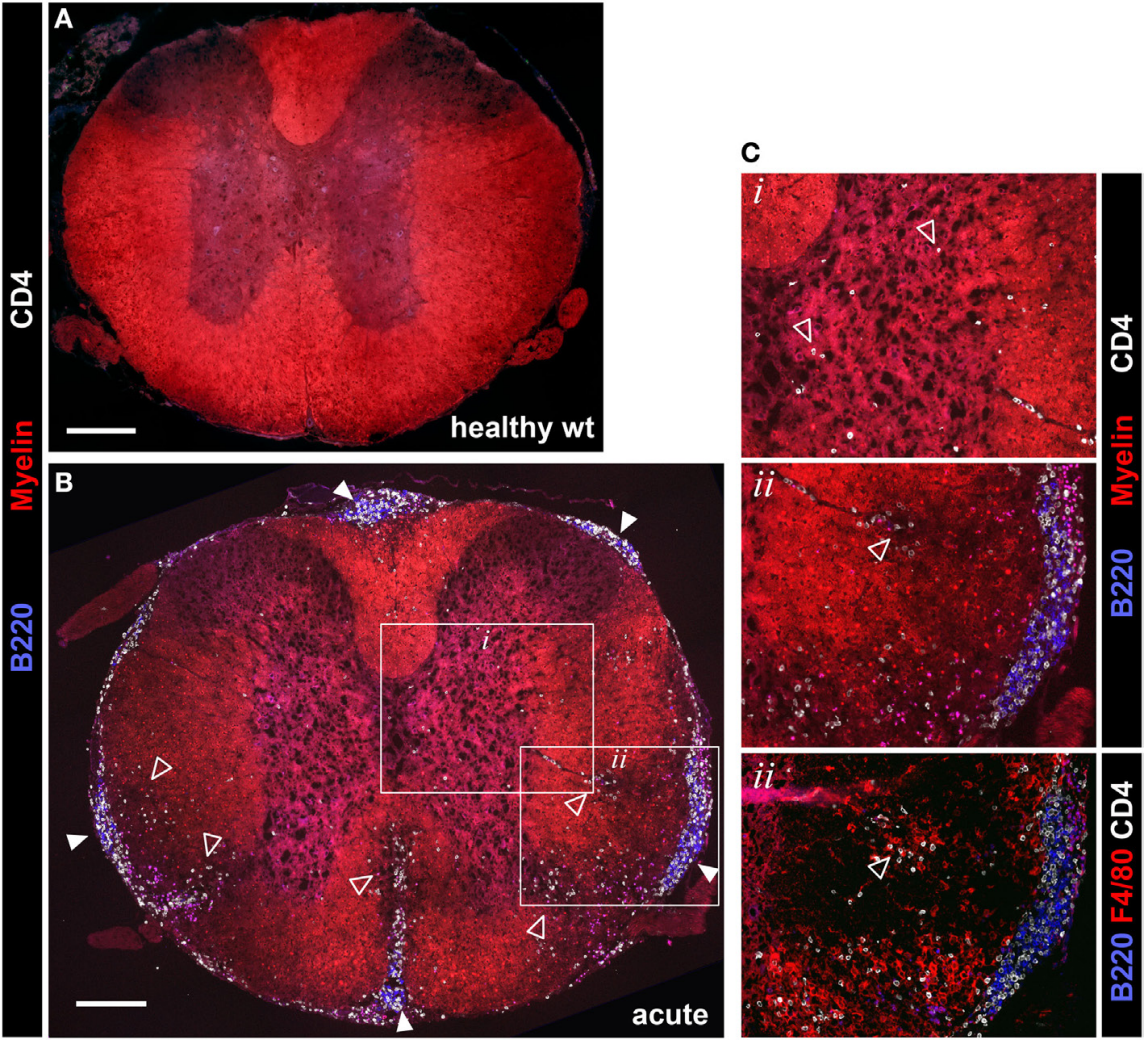
**Evaluation of spinal cord pathology in 2D2 IgH^MOG^ mice**. Mice were sacrificed in the acute phase of disease (<11 days post onset) and spinal cord pathology was evaluated by immunofluorescent histology. Sections were stained for myelin and invading CD4^+^ T cells and B220^+^ B cells. [**(A)** – wild type health control, **(B)** – 2D2 IgH^MOG^ acute disease]. Infiltrating T cells (open triangles) were evident in the gray matter of diseased mice [see **(B)** inset box *i*, shown at higher magnification in **(C)**, top panel]. Clusters containing B220^+^ B cells and CD4^+^ T cells (closed triangles) were clearly apparent in the meninges of diseased mice, while no B cells were found in healthy spinal cords [compare **(A)** with **(B)**]. Meningeal clusters were often adjacent to areas of demyelination and CD4^+^ T cell infiltration of the white matter (open triangles). Ongoing parenchymal invasion by T cells and macrophages/activated microglia were clearly evident, associated with regions of demyelination [open triangles, see enlarged image and serial section stained with F4/80, inset box ii, **(C)**]. Representative images shown (*n* = 5 wt, *n* = 5 acute phase 2D2 IgH^MOG^, minimum three sections taken from different regions of each spinal cord). Scale bars represent 200 μm.

2D2 IgH^MOG^ mice were sacrificed for histological evaluation of CNS pathology. No evidence of pathology or inflammation was evident in the CNS of wild type mice (Figure 4A) or 2D2 IgH^MOG^ mice that did not develop disease (not shown). Consistent with previous descriptions of this model (29, 30), there was little evidence of inflammation in the brains of 2D2 IgH^MOG^ mice that developed disease (not shown). By contrast, extensive and profound pathology was observed in the spinal cord. Evaluation of tissue harvested from mice in the acute phase of disease (<11 days post onset) revealed that extensive infiltration by CD4^+^ T cells (Figure 4, compare Figure 4A – wild type to Figure 4B – acute 2D2 IgH^MOG^) was associated with regions of reduced myelin staining (Figures 4B,C, inset box *ii*, middle) and F4/80^+^ macrophage/activated microglia (Figure 4C, bottom). CD4^+^ T cells were also observed in the gray matter in some mice and in these cases myelin staining of the gray matter was often altered compared to healthy mice (Figure 4C top, inset box *i*).

B cell infiltration of the spinal cord was almost exclusively restricted to the meninges although rare cells could be found in the white matter lesions. Meningeal B cells often formed clusters in close association with CD4^+^ T cells (Figures 4B,C, inset box *ii*) reminiscent of lymphoid clusters described in human MS tissue (3, 6, 22). Similar clusters were also reported in other investigations of this model (29, 30, 33), indicating that they are a consistent feature of disease in 2D2 IgH^MOG^ mice. These clusters were very often in direct association with regions of white matter demyelination and CD4^+^ T cell infiltration (Figure 4B). Pathology was not restricted to any particular region of the spinal cord as in some cases the entire spinal cord was involved, while in others inflammation was restricted to either distal (lumbar) or proximal (cervical) regions (not shown).

### B cell infiltration of the spinal cord is associated with chronic disease

**Figure 5:**
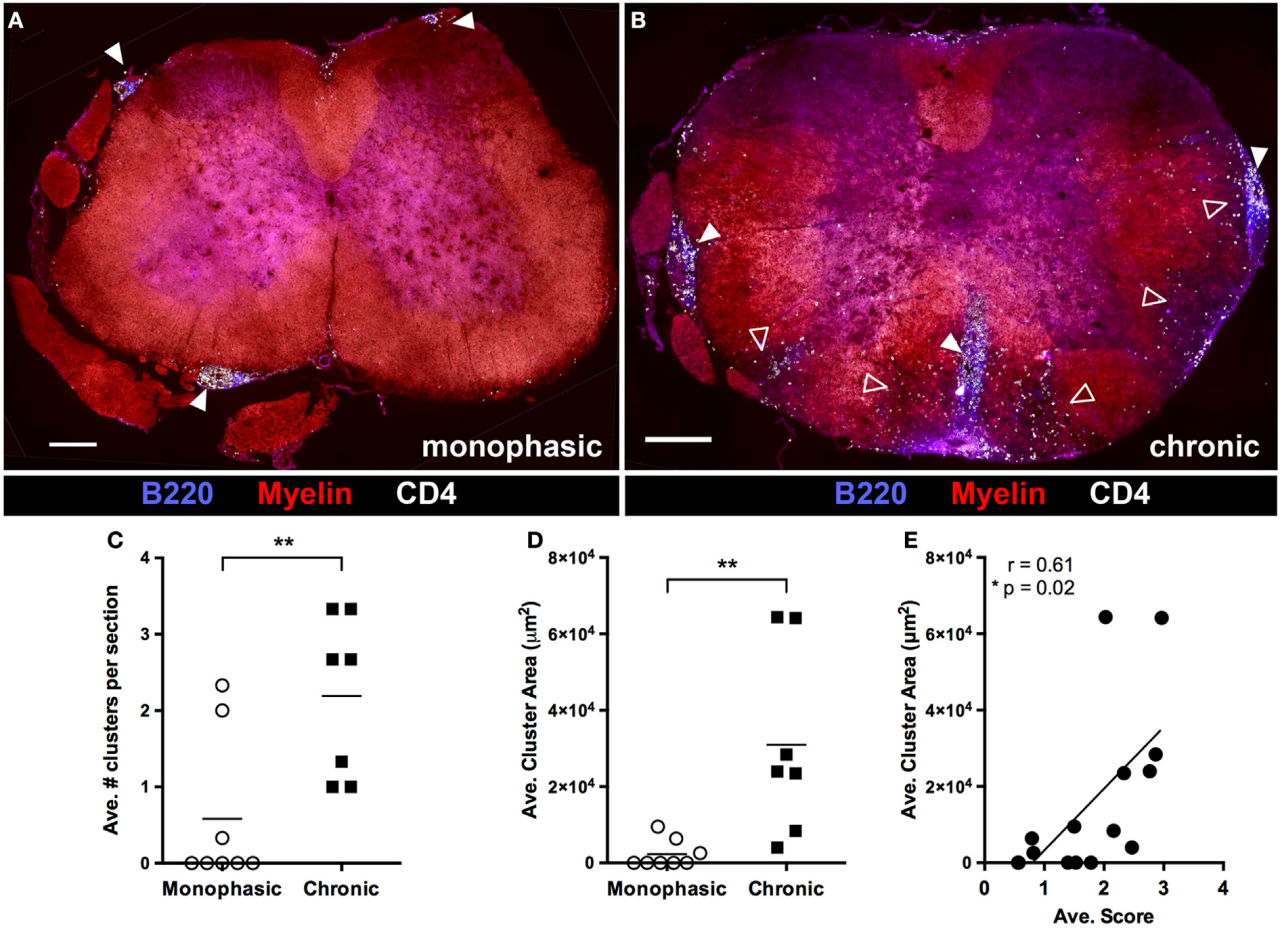
**B cell infiltration of the spinal cord is associated with chronic disease and increased disease severity**. At study endpoints (~4 weeks post disease onset), spinal cords were harvested for evaluation of pathology by immunofluorescence histology. Spinal cords from mice deemed to have either a “Monophasic” **(A)** or “Chonic” **(B)** disease course (as defined in Figure 2) were evaluated for demyelination, CD4^+^ T cell infiltration and meningeal cluster formation. Solid arrowheads indicate meningeal clusters. Open arrowheads indicate regions of white matter demyelination and infiltration by CD4^+^ cells. Scale bars represent 200 μm. The number of meningeal clusters per section **(C)** and cluster area **(D)** was evaluated using Image J software. Three sections from different regions of the spinal cord were evaluated from each mouse. **p < 0.01 as determined by Student’s *t*-test. Each symbol represents the average value per section from an individual mouse. **(E)** Cluster size was correlated with average disease score (as a measure of overall disease severity) for each individual mouse included in the study. Each symbol represents an individual mouse. A two tailed Pearson *r* test was performed to test for correlation.

We next evaluated spinal cord pathology later in disease (>20 days post disease onset) in mice with either a chronic or monophasic disease course (as defined above). White matter pathology in monophasic mice was very limited, in that there was little CD4^+^ T cell infiltration or demyelination (Figure 5A). By contrast, ongoing white and gray matter inflammation by CD4^+^ T cells and white matter demyelination was clearly evident in mice with chronic disease (Figure 5B) demonstrating that continued disability in these mice reflects active and ongoing inflammation, rather than permanent injury incurred during the initial attack. Large meningeal clusters containing T and B cells were also common in these mice. Nevertheless, despite reduced white matter involvement, small meningeal clusters were sometimes also present in monophasic mice (Figure 5A). However, subsequent analysis confirmed that meningeal clusters were both more numerous (Figure 5C) and larger (Figure 5D) in chronic vs. monophasic mice. Further, independent of disease course classification the size of meningeal clusters correlated with disease severity (Figure 5E). This, combined with the common spatial association between clusters and underlying regions of demyelination, suggests that these structures may contribute to ongoing chronic CNS autoimmune disease in 2D2 IgH^MOG^ mice.

### Characterization of B cells in meningeal clusters

**Figure 6:**
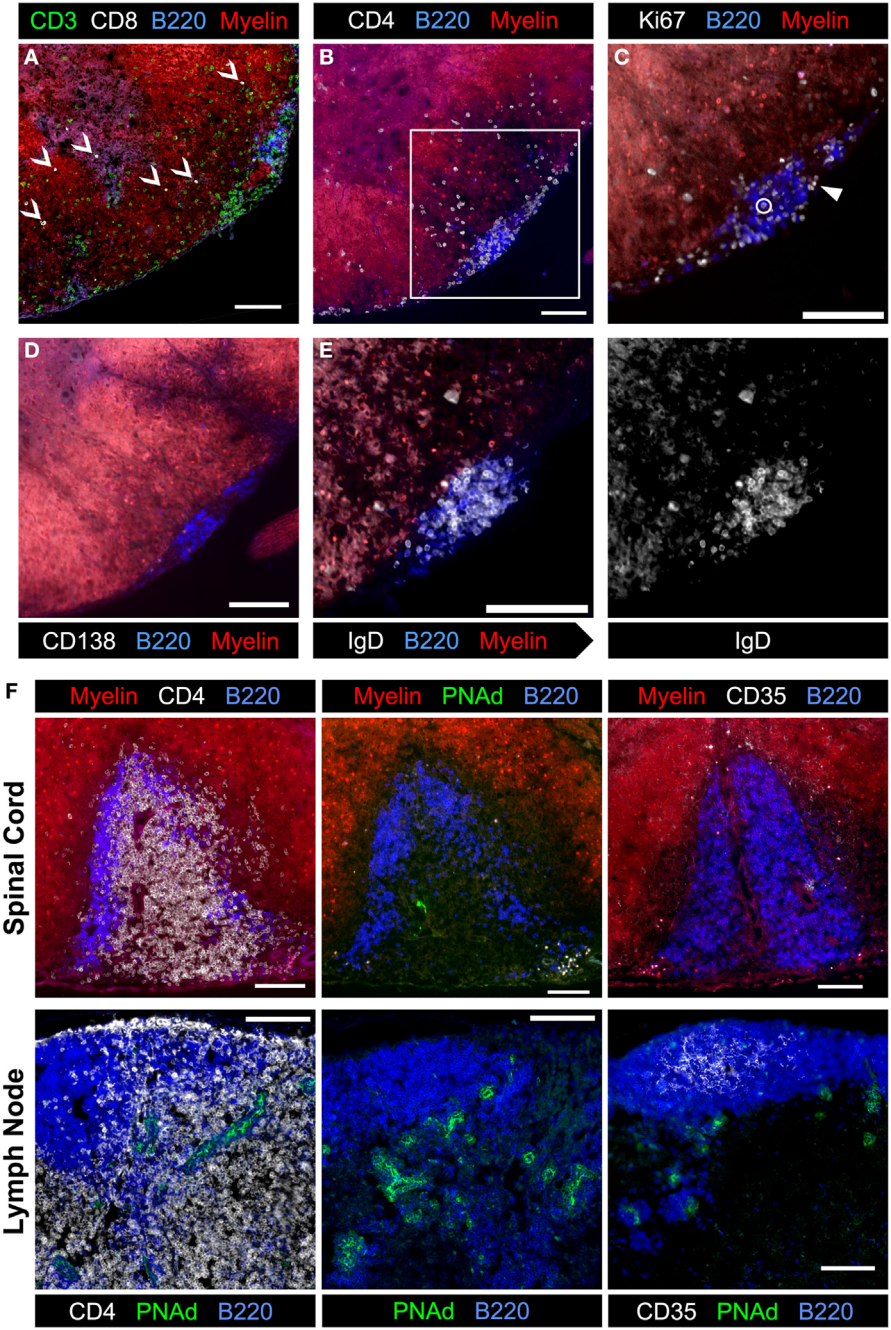
**Evaluation of meningeal clusters in spinal cords from 2D2 IgH^MOG^ mice with chronic sEAE**. Serial sections of spinal cord tissue from mice determined to have chronic disease (see Figure 2) were stained by immunofluorescence to characterize infiltrating immune cells. Images of one representative cluster from a single mouse (*n* = 7) are shown **(A–E)**. Scale bars represent 100 μm. **(A)** CD3^+^ CD8^+^ T cells were a common but minor component of the white matter infiltrate (open arrowheads), but only very rarely in meningeal clusters. **(B)** CD4^+^ cell infiltration into a region of demyelination adjacent to a meningeal cluster composed of B220^+^ B cells and CD4^+^ T cells. Inset box indicates the magnified region shown in subsequent serial sections. **(C)** Ki67^+^ cells in cell cycle were evident in meningeal clusters and in the affected white matter. The large majority of Ki67^+^ cells did not co-stain with B220 (example – closed arrowhead), with only very rare exceptions (open circle). **(D)** Little to no evidence of CD138^+^ plasma cells was observed in association with meningeal clusters. **(E)** Nearly, all B220^+^ B cells in meningeal clusters co-stained with IgD and therefore not class-switched (gray-scale of IgD channel alone shown on right). **(F)** Evaluation of meningeal clusters for evidence of features of lymphoid follicles. (Top) images of the single cluster from a 2D2 IgH^MOG^ mouse with chronic disease to show evidence of T and B cell organization into different separate regions (left, top – compare to B cell follicle and T cell zone separation in a healthy naïve lymph node, bottom) and differentiation of specialized high endothelial venules (middle top – compared to extensive PNAd staining in the lymph node, bottom). Little to no evidence of CD35^+^ follicular dendritic cells (right, top – compared to extensive follicular staining in the lymph node, bottom) was apparent in this meningeal cluster. For each stain listed above, between four and seven individual mice with chronic disease were evaluated, choosing sections with the most developed clusters.

To begin to dissect the role that B cells play in spinal cord pathology in sEAE, we evaluated the activation phenotype of infiltrating B cells. FACS analysis of lymphocytes isolated from spinal cords revealed that B cells are almost exclusively CD38^hi^ CD95^lo^, consistent with naïve or memory lymph node B cells (Figure 3A). However, compared to lymph node B cells with a similar CD38^hi^ CD95^lo^ phenotype, spinal cord B cells had significantly lower expression of CD62L and higher expression of CD80 (Figure 3C), indicating at least some level of non-classical activation, perhaps to present antigen. Cluster B cells were further characterized by histological examination of spinal cord tissue. We focused on spinal cords from chronic mice (see above) with evidence of ongoing disease activity. Consistent with a potential role for B cells in presenting antigen to T cells in clusters, T and B cells were found in close physical association with each other (Figures 6A,B). Subsequent staining confirmed that T cells in clusters were almost exclusively CD4^+^ T cells. However, we were surprised to find that CD8^+^ T cells were minor yet common component of the T cell infiltrate of white and gray matter (Figure 6A). This was not the case in the acute phase of disease (not shown). Although CD8^+^ T cells are known to infiltrate the CNS in human MS and contribute to some animal models of CNS autoimmunity (2, 35), we did not expect their presence in the 2D2 IgH^MOG^ model as the 2D2 TCR is derived from an MHC class II-restricted CD4^+^ T cell (26). However, CD8^+^ T cells were shown to infiltrate the CNS and participate in pathology in a similar model that makes use of a different MOG_35-55_-specific TCR on the NOD background. Although the TCR in this model was similarly derived from a CD4^+^ T cell, CD8^+^ T cells were found to express the transgenic TCR and recognize the MOG_35–55_ peptide presented on MHC class I (40).

We further investigated cluster B cells for evidence of activation. While Ki67^+^ cells were detectable within clusters as well as in the white matter, very few of them were co-stained with B cell markers (Figure 6B). Instead, the large majority of proliferating Ki67^+^ cells were T cells (not shown). CD138^+^ Plasma cells were not apparent in clusters (Figure 6C). Finally, cluster B cells were investigated for evidence of class switch. Virtually, all B cells expressed IgD (Figure 6D) and IgM, but not IgG1 (not shown).

It has been suggested that meningeal clusters in the CNS of MS patients may function as the so-called tertiary lymphoid tissues that recapitulate the structure and function of secondary lymphoid organs, such as lymph nodes and spleen (22). We therefore evaluated meningeal clusters in chronic 2D2 IgH^MOG^ mice for features of lymphoid follicles. With the exception of a single cluster in one mouse from the chronic group (Figure 6E), there was no evidence that T and B cells were organized into separate zones as occurs in lymphoid tissue (see Figures 4B, C, 5 and 6A–D for examples). Neither evidence of CD35^+^ follicular dendritic cells (FDCs) (Figure 6F) nor Bcl-6^+^ staining (GC B cells or T follicular helper Tfh cells) (not shown) was evident in any cluster that we examined. Finally, while some evidence of PNAd staining was apparent in the single cluster with evidence of T and B cell organization (Figure 6F), perhaps indicating development of specialized high endothelial venules, PNAd staining was not evident in any other cluster that we examined (not shown). Therefore, while it is possible that given sufficient time a proportion of meningeal clusters may attain some features of organized lymphoid tissue, the majority of clusters remain largely unorganized. If, as their association with demyelinating regions suggests, these clusters do contribute to the pathology of CNS autoimmunity, the less organized form must represent the minimum requirement for the pathogenic mechanism.

## Discussion

Here, we characterize a spontaneous model of CNS autoimmunity that depends on both T and B cell recognition of the myelin autoantigen. We are aware of only three previously published studies using this 2D2 IgH^MOG^ model. The original descriptions came from independent studies from Bettelli et al. (29) and Krishnamoorthy et al. (30) that focused principally on characterizing T cell activation as well as lesion distribution, which they found to be limited to the optic nerve and spinal cord. A third study made creative use of a version of this model to demonstrate that antibody production by MOG-specific B cells was not important to disease initiation, which instead was linked to antigen presentation by B cells to T cells (33). As discussed in more detail below, our findings presented here are largely consistent with these previous reports. We extend these studies by focusing on the B cell response and on characterizing infiltrating B cells and meningeal clusters in the diseased spinal cord.

The primary goal of our study was to characterize the B cell response in this B-cell-dependent model of CNS autoimmune disease. As expected, measurable GC responses were detected in lymph nodes from sick mice, although this was not true in all cases due to lymph node atrophy. Nevertheless, a GC response driven by interactions between MOG-specific T and B cells is presumably the mechanism for B cell-dependent disease initiation, as B cell presentation of antigen has been shown to be essential in a similar B cell-dependent mouse model (33). Of greater interest to therapeutic intervention in autoimmunity is the potential role of B cells in propagating ongoing disease, which may occur from within the inflamed CNS. With the exception of the involvement of CD8^+^ T cells, which were relatively common infiltrates of the spinal cord parenchyma in chronic but not acute disease, inflammation and pathology was qualitatively similar over the course of active disease. We observed extensive B cell infiltration of the meninges wherever white matter pathology was apparent, often forming clusters with CD4^+^ T cells. By contrast, white matter pathology was largely absent in mice that had recovered from monophasic disease. It is not clear if this reflects myelin repair or if extensive demyelination did not occur in these mice and disability was a reflection of inflammation, rather than actual tissue destruction.

Contrary to our initial expectations, B cells in meningeal clusters showed little indication of activation, with no evidence of class switch and little proliferation compared to infiltrating CD4^+^ T cells. By FACS, spinal cord B cells were CD38^hi^ CD95^lo^, consistent with naïve or memory cells, although elevated CD80 in particular suggests a degree of activation. Further investigation will be required to determine if B cells upregulate CD80 once in the meninges or if CD80^hi^ B cells are selectively recruited. It should be noted that B cell follicles in secondary lymphoid tissues are largely populated by naïve B cells, and therefore an unactivated phenotype would be expected if meningeal clusters do indeed represent lymphoid structures. However, with the possible exception of the single cluster in a mouse with chronic disease described above, we did not observed any other evidence of typical follicular features in the meningeal clusters that formed in sick 2D2 IgH^MOG^ mice. We previously observed similar clusters in a model of EAE induced by immunization with a protein antigen based on mouse MOG (25). Again, there was no evidence of follicular differentiation in these clusters. Together, these models suggest that cluster formation is common in models that incorporate B cell recognition of the autoantigen. This is not an absolute requirement, however, as small clusters could still form in mice with mutant BCR incapable of recognizing MOG or in mice immunized with the standard short MOG_35–55_ peptide (25). White matter demyelination and inflammation were much reduced compared to what we observed in either MOG protein-induced disease or in sick 2D2 IgH^MOG^ mice, which also feature greater meningeal B cell infiltration.

Models of CNS autoimmunity that incorporate target recognition by more than just CD4^+^ T cells, such as 2D2 IgH^MOG^ mice or EAE models induced with protein antigen, represent significant improvements over peptide-induced models that are used most commonly today as they allow for a more normal and complex response. Responses to “real” antigens recruit multiple immune targeting and effector processes. Nevertheless, it is not clear that these models more accurately represent human MS. Focusing specifically on investigations of infiltrating B cells in MS, lineage analysis suggest that B cells isolated from CNS represent a population derived from the GC response and subsequently selected from the peripheral pool (4, 41, 42). FACS studies suggest that CSF B cells are enriched for CD27^+^ cells (4, 43, 44), which in humans is considered a marker of memory. Therefore, these studies suggest that at least some B cells in human disease were previously activated. As there is no equivalent memory marker in mice, it is not currently possible to directly compare these observations to our own of the 2D2 IgH^MOG^ model.

Histological studies of human post-mortem tissue have been inconsistent in finding meningeal clusters (3, 6, 45–48). When clusters have been observed (3, 6, 45), more examples of follicular differentiation were reported than we observed in our mouse models [here and in Ref. (25)]. Nevertheless, our observations suggest that, given time, it is possible that meningeal clusters can attain at least some features of true follicles. Human studies were performed on tissues from patients who had disease for many years and in most cases decades (22), and despite their focus on the most follicle-like structures, it is clear that most meningeal clusters in MS remained largely unorganized (22, 23), consistent with our observations in both mouse models. Furthermore, human studies were almost exclusively of progressive disease, where neurodegeneration occurs even though there is less evidence of active inflammation (2). It is not clear that these observations are relevant to the earlier active inflammatory stage of disease that is likely a better correlate to EAE. The rarity of tissue from this earlier stage of disease and of spinal cord tissue will make direct comparison very difficult. Nevertheless, as the best evidence supports the hypothesis that MS is an autoimmune disease targeting myelin antigens, holistic models of CNS autoimmune disease that more completely involve the immune system have the best chance of revealing important fundamental pathogenic B cell mechanisms that drive ongoing disease.

The apparent contradictory effects of B cell depletion by anti-CD20 (17) vs. TACI-Fc (18) in human MS highlights the urgent need to better understand the complex biology of these cells in autoimmune disease and the immune response in general. Both anti-CD20 and TACI-Fc target IL-10-producing Breg cells (19, 49). However, they have very different activity on plasma cells and therefore antibody production (19), as well as T cell biology (20). There are likely additional B cell subsets with differential susceptibility to depletion by each reagent. Indeed, IgM^+^ memory B cells are more dependent on BAFF and therefore to depletion by TACI-Fc than class-switched memory cells (50). Anatomical location may also affect susceptibility to depletion by either reagent. Work to decipher this will have to rely heavily on models, such as the 2D2 IgH^MOG^ mice, as in human patients usually only the circulating and more rarely CSF pools can be accessed. Our models suggest that antigen-specific B cells contribute to disease but that, unexpectedly, the B cell infiltrate in the CNS are not activated in a way that we would expect based on studies of antigen-specific activation in lymphatic tissue. Further work will be required to identify subsets of B cells, how they contribute to pathology or protection from disease, and their susceptibility to different methods of therapeutic intervention.

## Conflict of Interest Statement

The authors declare that the research was conducted in the absence of any commercial or financial relationships that could be construed as a potential conflict of interest.
